# Field effectiveness of microbial larvicides on mosquito larvae in malaria areas of Botswana and Zimbabwe

**DOI:** 10.1186/s12936-016-1642-6

**Published:** 2016-12-06

**Authors:** Mulamuli Mpofu, Piet Becker, Kaka Mudambo, Christiaan de Jager

**Affiliations:** 1 Faculty of Health Sciences, School of Health Systems and Public Health, University of Pretoria Institute for Sustainable Malaria Control (UP ISMC), University of Pretoria, 31 Bophelo Road, Gezina, Pretoria, South Africa; 2 Southern African Regional Network (SARN), Roll Back Malaria Partnership, East and Southern Africa Secretariat, Gaborone, Botswana

**Keywords:** Larviciding, Microbial larvicides, Botswana, Zimbabwe, Malaria vector control

## Abstract

**Background:**

The successful control of malaria vectors requires the control of both the larval and adult stages. The adult control methods through indoor residual spraying (IRS) and use of long-lasting insecticidal nets (LLINs) continue to be widely used with some high measure of success. Larval control methods are also being used by a number of National Malaria Control Programmes (NMCPs) with limited understanding of its contribution. Larval control might be needed in some areas to move from malaria control to elimination. This experimental study was conducted to assess the field effectiveness of winter larviciding on the larval stages of the mosquito in Botswana and Zimbabwe.

**Methods:**

Two villages were selected in each of the two countries, one as an intervention and the other as the control. Water bodies in the intervention villages were treated using the commercial product VectoBac^®^ WG (Valent BioSciences Corporation, IL, USA) containing the active ingredient *Bacillus thuringiensis* var. *israelensis* (*Bti*), a WHO recommended bio-larvicide, applied at a rate of 300 g per hectare. Random-effects Poisson regression was employed during data analysis to compare intervention with control sites with respect to larval counts.

**Results:**

The average marginal effect of larviciding on the mosquito larvae taking interaction with time (period) into account, was −1.94 (95% CI −2.42 to −1.46) with incidence rate ratio of 0.14, thus an 86% larval reduction attributable to the intervention for both countries combined. There was a 92% and 65% effect for Botswana and Zimbabwe respectively. The effect on the early larval and late stages was 77% (P < 0.001) and 91% (P < 0.001), respectively. Overall, intervention larval sampling points had five more larvae than the control at baseline and 26 less after 16 weeks. The effect on the different species also showed similar trends.

**Discussion/conclusion:**

Larval control using *Bti* showed a high effect on the population of the mosquito larvae. The reduction of the early and late larval stages can lead to reduced adult mosquito emergence and low adult mosquito densities. Larviciding can be used to control mosquito vector population by suppressing the larval stages thereby reducing adult emergence and malaria risk.

## Background

The World Health Organization (WHO) has targeted malaria for elimination which can be achieved through strengthening of country surveillance, diagnosis, case management and vector control activities [1, 2]. Implementation of proven vector control interventions of long-lasting insecticide-treated nets (LLINs) and indoor residual spraying (IRS) is currently at the core of successful malaria vector control [3–5]. LLINs protect their occupants by diverting host-seeking vectors and by killing those that attempt to feed [6, 7], but the number distributed annually since 2005 has remained below the required number to reach universal access [8–10]. In sub-Saharan Africa early malarial eradication pilot projects showed that malaria is highly responsive to vector control by IRS [11, 12], with the first trial being carried out in 1931 in KwaZulu-Natal, South Africa using pyrethrum. In the 1950s, IRS with DDT had become the main vector control method in South Africa [11].

Integrated vector management (IVM), targeting both larval and adult mosquitoes has lately received a lot of attention because of its potential in control and elimination of malaria [4, 5]. The interest in larval control led the WHO to issue an *Interim Position Statement on Larviciding in sub*-*Saharan Africa* in 2012 [3]; and subsequently developed and launched the *Larval Source Management (LSM) guidelines* in 2013 [13]. Recent evidence of larviciding effectiveness presents an opportunity in sub-Saharan Africa, particularly in urban areas because of the rapidly growing urban centres [14]. Studies have shown effectiveness of larviciding in urban areas [14–17], in highlands [18], and when used in combination with LLINs [3–5]. Based on available data, larviciding has been recommended as a complementary intervention to IRS and LLINs [13], and to be utilized in areas with water bodies which are few, fixed and findable while additional research is being conducted to measure the effectiveness of the intervention [19].

Reports received from national programmes indicate that 48 malaria-endemic countries worldwide use larval control in certain specific foci of malaria transmission of which 18 are in sub-Saharan Africa [10]. These reports give an indication of the range of larval control methods employed, but the scale of efforts is not quantified and the impact on individual country malaria burden is not easily measured. With the increasing trends of resistance to pyrethroids used for IRS and for treating LLINs [20–24], there is a demand for alternative technology and products to help in the control and subsequent elimination of malaria. Behavioural adaptation of adult mosquito vectors gives them the ability to avoid LLINs and walls treated through IRS [25–28], while research on other potential malaria control interventions such as transmission-blocking vaccines and genetically modified mosquitoes have not been successful [29]. There is current discussion that relying solely on IRS and LLINs may be insufficient to achieve malaria elimination in much of sub-Saharan Africa [15], and larviciding would have to be part of an integrated vector management (IVM) approach [30] that could help hinder malaria transmission.

This study was conducted in selected semi-arid rural areas of Botswana and Zimbabwe to establish the effectiveness of winter larviciding as an additional vector control intervention. The study involved assessment of the effectiveness of larviciding on larval density as well as adult mosquito density. This paper, presents a comprehensive analysis of the effectiveness of winter larviciding on larval density in two semi-arid regions.

## Methods

### Study design and setting

An experimental study was conducted in two neighbouring countries, Botswana and Zimbabwe. In Botswana, Mathathane and Molalatau villages in Bobirwa District, which are 25 km apart were selected as the intervention and the control villages, respectively. In Zimbabwe, Birchenough Bridge which is an irrigation area was used as the study village, with the northern part of the village being used as the intervention area and the section of the village south of the irrigation scheme being used as the control. The irrigation fields acted as the buffer between the intervention and the control area, and distance between the two study arms was 5 km. With north to south bound winds, there was minimal expectation that mosquitoes will fly from the control areas to the intervention area.

The villages in the two countries were selected with the help of the national malaria control programmes for the assessment of larviciding in supporting elimination efforts in similar localities. The whole of Botswana is in the pre-elimination phase and experiencing concentrated malaria with low transmission. Mathathane and Molalatau villages in Botswana have previously reported sporadic malaria outbreaks and entomological surveys have yielded positively on malaria vectors. In Zimbabwe, Birchenough Bridge which is in Buhera District, reports the lowest number of malaria cases in Manicaland province and has the potential to be considered for malaria pre-elimination.

### Data collection

#### Larval habitat surveying and mapping

The location and type of larval breeding habitats was surveyed in May 2015 in both the intervention and the control villages. All semi-permanent and permanent aquatic mosquito habitats in the intervention and control villages were mapped using handheld geographic positioning system receivers. Mapped habitats were given a unique identification name and number to allow quick reference during field operations, including global positioning system (GPS) coordinates [13].

In both countries, the study arms were characterized by few larval habitats as all of the temporary habitats had dried up. Breeding was mainly along river beds, with a lot of animal activity which was creating thousands of minute breeding points. However, these hoofmarks were not mapped as separate breeding points.

In the intervention area of Zimbabwe, larval habitats were tributaries draining from Save river and the irrigation area through seepage. The two tributaries referred as Bonda Mud and Bonda Sand permanently have water throughout the year, each stretching for almost 500 m. At the start of the data collection, the two tributaries including their collection ponds had an estimated combined water surface area of 0.5 hectares with bonda sand being approximately 0.3 hectares and bonda mud being 0.2 hectares. In Zimbabwe, the control area was downstream and south of the irrigation. Permanent breeding was along five irrigation drains feeding into a stream that later drains into the Save river a further three kilometres from the human settlements. Additional breeding was along a drain from a borehole that supplies potable water to the local residents.

In Mathathane, the intervention village in Botswana, breeding occurred along river beds of Selepye and Mathathane that pass through the village. The village lies on an aquifer and on the southern part of the village water naturally comes out, flows and settles along the beds of the two rivers. It flows and covers a distance of 400 m along Mathathane river with a water surface area of approximately 0.5 hectares while along Selepye river it covers a surface of approximately 1.5 hectares along its 2 km stretch. In the control village of Molalatau, the water source to the river where permanent breeding occurs is the borehole drilled on the aquifer where water flows out under pressure into the river.

#### Treatment of larval habitats

Treatment of the larval habitats was done in winter of 2015, from June to October, using the commercial product VectoBac^®^ WG (Valent BioSciences Corporation, IL, USA) containing the active ingredient *Bacillus thuringiensis* var. *israelensis* (*Bti*), a WHO recommended bio-larvicide [31]. Application of biolarvicide was conducted in intervention areas/villages at two week intervals for eight time periods. Though mapped, water bodies in the control villages were not treated. In both Botswana and Zimbabwe, larviciding was conducted by community volunteers who were identified with the help of the local community leadership and through the local health facilities.

In Zimbabwe, two community volunteers participated in the study and worked under the full supervision of an entomologist seconded to the study by the Ministry of Health. In Botswana, three community volunteers participated throughout the study. The main responsibilities of the community volunteers was to identify breeding habitats, conduct larviciding and assist the entomologist in larval sampling. They received training from the study coordinator and the entomologist on how to identify breeding sites and complete the habitat survey forms and on how to apply larvicide. Through their interaction with the entomologist and the study coordinator, they also learnt how to sample larvae and determine larval density by type and stage. However these activities were the responsibility of the study coordinator and the entomologists, and the volunteers were conducting these activities under supervision. Knap sack sprayers were used for application of the larvicide at a rate of 300 grams per hectare surface of water.

#### Intervention timelines

**Fig. 1:**
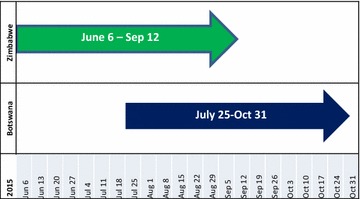
Intervention timelines for Botswana and Zimbabwe

Implementation of the intervention started early June in Zimbabwe (Fig. 1) and seven weeks later in Botswana. Implementation continued for 16 weeks at each of the two countries at two week intervals.

#### Entomological surveys

Larval inspections and sampling was conducted consistently at two week intervals before the next treatment in intervention areas, and the next day in the control villages. VectoBac^®^ WG product has previously demonstrated to have a high effectiveness within the first 24–48 h on low doses [32]. In this study, larval sampling was repeatedly conducted at the same sampling points, and 14 days after treatment and just before the next treatment. During larval sampling, data on both habitat occupancy and larval density was collected.

##### Habitat occupancy

The presence or absence of larvae in a breeding site was determined by visual observation. If a habitat was positive (i.e. larvae are present), the next step was to determine larval density.

##### Larval density

The presence or absence of larvae was scored after a minimum of 10 dips per site [33], taken with a standard 250 ml capacity mosquito dipper (Clarke Corporation, IL, USA). The following information was recorded for every site during the surveys: (i) the presence or absence of early larvae stages (stages I and II instars), (ii) late stages (stages III and IV instars). The larvae were also disaggregated by specie, either *Anopheles* or *Culex*. The proportion of late instar larvae was calculated as an indicator of larval survival, adult mosquito emergence and appropriateness of application by the larvicider. Morphological features used to identify *Culex* larvae were a rounded head, presence of a long siphon tube, and a resting position which is at an angle to the water surface. *Anopheles* features included a long head, a short and at times invisible siphon tube, and a resting position which is parallel to the water surface.

### Statistical analyses

From each larval sampling site, larval counts at each of eight time periods 14 days apart were done. The time periods for the two countries did not coincide exactly, the Zimbabwe arm ran for periods one through eight and the Botswana arm from period four to 11. The analyses assessed larval counts for intervention and control sites. Random-effects Poisson regression was employed to assess the relationship between larval counts and the fixed-effects treatment (larvicided; not larvicided), country (Botswana; Zimbabwe), time period, the interaction between treatment and time period and covariate baseline count. Sites were specified as the random-effects component with an intercept and takes care of the repeated measures within sites. The incidence rate ratio (IRR) for treatment was of primary importance. Data analysis was done using Stata Release 13 and14, (StataCorp, College Station, TX: StataCorp LP).

### Ethical considerations

Approval for the study was obtained from the Ethics Committee of the Faculty of Health Sciences, University of Pretoria (South Africa, Protocol number 289/2014); The Human Research Development Committee (HRDC) of the Botswana Ministry of Health; and the Medical Research Council of Zimbabwe (Approval number MRCZ/A/1898). Support to conduct the study was obtained from the National Malaria Control Programmes of both Zimbabwe and Botswana. Local chiefs were used as the entry point to the study communities and community consent for the implementation of mosquito larval control was obtained during community meetings initiated through the local area chief and village heads.

## Results

### Larval density

Table 1 below shows the average larval density per dip at visit one through visit eight in both Botswana and Zimbabwe. Data collection started early in Zimbabwe on the 4th of June 2015, ending on 12th of September 2015, while in Botswana data collection and the interventions started on the 25th of July 2015 and ending on the 30th of October 2015.

**Table 1:** Larval density by visit and country

	Average number of larvae per dip by country and visit					
	Botswana			Zimbabwe		
	Calendar date (2015)	Intervention	Control	Calendar date (2015)	Intervention	Control
Visit 1	July 25	32	33	June 6	22	10
Visit 2	Aug 8	16	32	June 20	16	3
Visit 3	Aug 22	3	36	July 4	7	8
Visit 4	Sept 5	2	35	July 18	5	3
Visit 5	Sept 19	3	32	Aug 1	4	5
Visit 6	Oct 3	3	32	Aug 15	1	14
Visit 7	Oct 17	1	31	Aug 29	3	25
Visit 8	Oct 31	2	32	Sept 12	2	22

*NB* The average number of larvae was rounded to the next whole number

Botswana started with high larval counts for both intervention and control which were comparable. On average, there were 32 and 33 larvae per dip in the intervention and control area respectively. Zimbabwe had an average larval count of 22 per dip at baseline (visit 1) in the intervention area, and 10 larvae in the control. The control areas in Botswana maintained larval density at more than 30 while in Zimbabwe, there was a reduction to an average of three larvae. These counts are the average for all the sampling points in each of the two countries and study arms.

### Average change in larval density

**Fig. 2:**
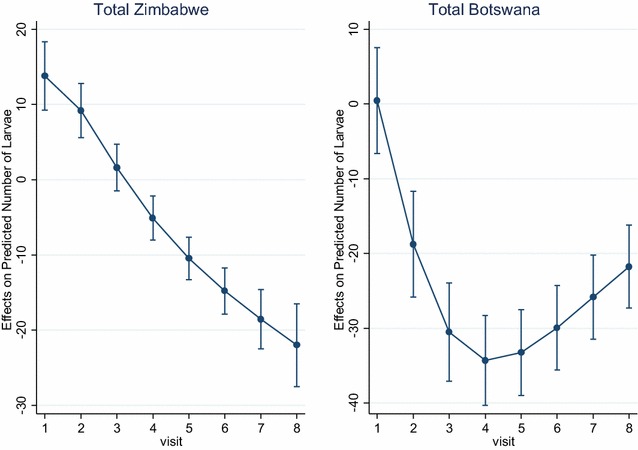
Average marginal change from baseline in larval counts between intervention and control for Zimbabwe and Botswana

Table 2 and Fig. 2 show the predicted difference/change in number of larvae in the intervention areas relative to the control by visit and country attributable to larviciding.

**Table 2:** Average marginal effects (CI) in larval counts between intervention and control

Visit	Botswana	Zimbabwe
	Marginal effect (CI)	Marginal effect (CI)
Visit 1	0.45 (−6.61; 7.52)	13.77 (9.22; 18.33)
Visit 2	−18.76 (−25.83; 11.69)	9.20 (5.60; 12.81)
Visit 3	−30.53 (−37.10; 23.96)	1.61 (−1.47; 4.70)
Visit 4	−34.29 (−40.30; 28.27)	−5.09 (−8.01; −2.18)
Visit 5	−33.25 (−38.98; 27.51)	−10.45 (−13.27; 7.63)
Visit 6	−29.93 (−35.60; 24.26)	−14.80 (−17.86; 11.75)
Visit 7	−25.82 (−31.44; 20.19)	−18.56 (−22.50; 14.62)
Visit 8	−21.76 (−27.31; 16.21)	−22.01 (−27.54; 16.48)

Marginal effect for factor levels is the discrete change from the base level

From Table 2 above, the average difference in the total number of larvae as a result of intervention ranged from 0.45 at visit one to 21.76 at visit eight, and 13.77 and 22.01 for Botswana and Zimbabwe, respectively. At baseline or visit 1, the intervention and control sites in Botswana were comparable with an average difference in number of larvae of less than one, while in Zimbabwe intervention sites had an average of 13.77 more larvae relative to the number in the control though not statistically significant. From the analysis, the effect of the intervention demonstrated a reduction in larval density in the intervention areas of Botswana relative to the control, while in Zimbabwe the predicted effect through visits is rather an increase in larval population in the control areas, while the intervention remained stable and low due to the introduction of larviciding.

From Fig. 2 above, there was a steady increase in change from baseline in larval density between the intervention and the control over time in Zimbabwe. In Botswana, there was a sharp increase in change from baseline after the introduction of the intervention, which then evened out from visit 5 until the end of data collection at visit 8.

### Effect of intervention on larvae

Table 3 below shows the marginal effect of larviciding on the larvae, its effect on the different larval stages and the larval specie, as well as the incidence rate ratio.

**Table 3:** Marginal effect of larviciding in Zimbabwe and Botswana

Larvae type	Marginal effect	IRR (CI)	P value	% reduction
*All larvae*				
Both countries	−1.94	0.14 (0.09; 0.23)	<0.001	86
Zimbabwe	−1.06	0.35 (0.24; 0.51)	<0.001	65
Botswana	−2.51	0.08 (0.06; 0.11)	<0.001	92
***Larval stage***				
*Early instar*				
Both countries	−1.47	0.23 (0.17; 0.32)	<0.001	77
Zimbabwe	−1.23	0.29 (0.17; 0.50)	<0.001	71
Botswana	−1.83	0.16 (0.12; 0.22)	<0.001	84
*Late instar*				
Both countries	−2.40	0.09 (0.04; 0.19)	<0.001	91
Zimbabwe	−0.56	0.57 (0.31; 1.03)	0.062	43
Botswana	−4.04	0.02 (0.01; 0.04)	<0.001	98
***Larvae species***				
*ALL Anopheles*				
Both countries	−1.63	0.20 (0.05; 0.76)	0.019	80
Zimbabwe	−0.64	0.53 (0.30; 0.92)	<0.025	47
Botswana	−3.01	0.05 (0.02; 0.13)	<0.001	95
*ALL Culex*				
Both countries	−1.03	0.36 (0.10; 1.23)	0.102	64
Zimbabwe	−0.86	0.42 (0.26; 0.68)	<0.001	58
Botswana	−2.21	0.11 (0.05; 0.27)	<0.001	89

The average marginal effect of larviciding on the mosquito larvae, taking interaction with time (period) into account, was −1.94 (95% CI −2.42 to −1.46) with incidence rate ratio of 0.14, thus an 86% larval reduction attributable to the intervention for both countries combined. There was a 92 and 65% effect for Botswana and Zimbabwe respectively. The reduction (%) on the early and late larval stages was 77% (P < 0.001) and 91% (P < 0.001) respectively (Table 3).

The effects of larviciding were also significant for the different larval species, with 95% and 47% reduction of *Anopheles* larvae for Botswana (P < 0.001) and Zimbabwe (P = 0.025), respectively. The seemingly low reduction in Zimbabwe is due to small denominators and numerators because of low breeding activity during winter. The average marginal effects on the *Culex* larvae were −2.21 (89% reduction) and −0.86 (58% reduction) for Botswana and Zimbabwe, and were both statistically significant (P < 0.001).

## Discussion

The study has demonstrated that larviciding using *Bti* is an effective vector control intervention in low transmission malaria areas of Botswana and Zimbabwe because of the reduction of the larval stages of mosquito. *Bti* functions as a stomach poison in the mosquito larval midgut, and its effect on larvae is largely due to protoxins in parasporal crystals and the spore coat, rather than the actual infection [34, 35], and is usually active for one to two weeks generally requiring fairly clean water to be effective [35, 36]. All treated water bodies in the intervention areas of both Botswana and Zimbabwe were fresh water points, with minimal pollution due to animal activity, and larviciding happening every two weeks. *Bacillus thuringiensis* var. *israelensis* and *Bacillus sphaericus* based microbial larvicide products have lately been assessed and found to be effective in reducing malaria vector mosquito larvae under field conditions, and subsequent reduction in malaria vector population densities [32, 37–40]. Efficacy trials have also illustrated that *Bti* can reduce malaria transmission when implemented at a large scale [14], and when delivered as a supplementary measure alongside LLINs [5].

While most field studies have been conducted in highlands and urban areas [5, 14, 41, 42], this is probably the first field study in semi-arid malaria transmission areas of sub-Saharan Africa, experiencing low transmission and in pre-elimination of malaria. The demonstrated effectiveness of *Bti* in inhibiting the progression from the early stages to the late larval stages is an indicator on its effect on adult mosquito emergence and risk of malaria transmission. In the control areas, larval development progressed uninterrupted to the late stages, while larviciding effect on the more sensitive early instars resulted in fewer larvae progressing to the late stages. Studies have shown that early instars are more susceptible than the late instars to various formulations of *Bti* [43, 44], which reduces the occurrence of the later during habitat treatment periods [33].

Implementation of larviciding started at the beginning of winter in Zimbabwe, and later towards the end of winter in Botswana. While the study areas in the two countries have comparable environmental conditions, larval density was lower at baseline in Zimbabwe compared to Botswana. This is understandable considering that breeding activity is usually lower when temperatures are at their lowest early in winter. However, despite the different levels at baseline, the intervention showed high effectiveness at first treatment, which was sustained. For Botswana, later during the study, the difference in the larval density in the intervention and the control reduced due to an increase in larval density in the intervention. Since the intervention continued to be implemented at 14 day intervals despite increasing temperatures, this could be due to the effect of rising temperatures on the microbial larvicides. At high temperatures, solar inactivation has been found to affect microbial larvicidal products [41]. Elsewhere, *Bti* has shown a residual effect of up to 10 days in standardized field tests implemented during the dry season, providing complete protection when applied weekly [44]. Low doses of 200 g/ha is required to effectively suppress late instars, but can also lead to the absence of residual activity [45]. For this study, doses of 300 g/ha were applied. Despite the start of the intervention at different times of the year in the two countries, *Bti* still demonstrated a significant effect on the larval population when comparing the intervention and the control areas in both countries.

The effectiveness of *Bti* in both Botswana and Zimbabwe was consistent for both the *Anopheles* and *Culex* larva, and for the different stages of both species. While the *Culex* species are not vectors of human malaria, the reduction in its population reduces human exposure to mosquito bites which is also important in the general population’s assessment of the effectiveness of a malaria vector control intervention. *Bti* formulations are known to have a large activity spectra covering larvae from many Culicidae (mosquito) genera: *Culex*, *Aedes*, and *Anopheles* [43, 44, 46], which reduces overall adult mosquito emergence and human exposure to bites and transmission [5, 33].

## Conclusion

The use of the microbial larvicide *Bti* has shown to have an impact on larval densities in low malaria transmission areas of Botswana and Zimbabwe, which can lead to a reduction in adult mosquito densities and malaria transmission. The results of this study presents an opportunity for strengthening integrated vector management by including larviciding. Mosquito larvae, unlike adults are relatively immobile and cannot change their habitat to avoid control activities making larviciding an effective vector control intervention which can be used in semi-arid malaria areas with low transmission such as Botswana and parts of Zimbabwe. It can be considered as an additional intervention for malaria elimination in such areas.

## Abbreviations

LLINs: long-lasting insecticide-treated nets
NMCP: National Malaria Control Programme
*Bti*: *Bacillus thuringiensis var. israelensis*
WHO: World Health Organization
IRS: indoor residual spraying
DDT: Dichloro-diphenyl-trichloroethane
IVM: integrated vector management
